# Skilled Nursing Facility Changes in Ownership and Short-Stay Medicare Patient Outcomes

**DOI:** 10.1001/jamanetworkopen.2023.34551

**Published:** 2023-09-19

**Authors:** Rachel A. Prusynski, Andrew Humbert, Tracy M. Mroz

**Affiliations:** 1 University of Washington Department of Rehabilitation Medicine, Seattle

## Abstract

**Importance:**

Skilled nursing facility (SNF) changes in ownership are receiving attention in the national conversation regarding health care quality and oversight. SNF ownership changes have been cited as possible ways for SNFs to obscure financial arrangements and shift funds away from patient care; however, it is unclear whether ownership changes are associated with quality outcomes, especially for short-stay patients.

**Objective:**

To determine which SNF characteristics are associated with changes in ownership and whether ownership changes were associated with differences in short-stay patient outcomes.

**Design, Setting, and Participants:**

This cohort study was a secondary analysis of 2016 to 2019 Medicare administrative data including SNFs in the United States with complete data. Data were analyzed from January 2016 through December 2019.

**Exposure:**

SNF change in ownership.

**Main Outcomes and Measures:**

Outcomes of interest were facility-level risk-adjusted rates of hospital readmissions, emergency department visits, and community discharge for short-stay patients after admission to an SNF. Analyses were conducted using multivariable logistic regression and controlled interrupted time series.

**Results:**

Of 11 004 SNFs, 1459 (13.26%) changed ownership between 2016 and 2019. Compared with for-profit SNFs, nonprofit and government SNFs had lower odds of changing ownership (nonprofit: odds ratio [OR], 0.40; 95% CI, 0.32-0.49; government: OR, 0.26; 95% CI, 0.17-0.41). Chain SNFs had higher odds of changing ownership than nonchain SNFs (OR, 1.38; 95% CI, 1.21-1.59). Urban SNFs with lower occupancy rates (OR per 10–percentage-point decrease, 1.19; 95% CI, 1.14-1.25), larger Medicaid populations (OR per 10–percentage-point increase, 1.17; 95% CI, 1.13-1.22), and lower staffing ratings (OR per 1-star increase on staffing rating, 1.18; 95% CI, 1.14-1.25) had higher odds of changing ownership. Descriptively, all 3 quality outcomes were worse throughout the study in SNFs undergoing ownership change compared with controls that did not change ownership. However, results of interrupted time series models found no associations between an ownership change and hospital readmissions or community discharge rates. Ownership change was associated with a short-term increase of 0.32 (95% CI, 0.03 to 0.62) percentage points in emergency department visits.

**Conclusions and Relevance:**

In this cohort study of 11 004 SNFs in the US between 2016 and 2019, SNF characteristics historically associated with lower quality were more likely to change ownership; however, ownership changes were only associated with short-term increases in ED visits. These results suggest that SNF ownership changes may be a symptom, not a cause, of lower quality.

## Key Points

**Question:**

What types of skilled nursing facilities (SNF) change ownership, and are SNF sales associated with short-stay patient outcomes?

**Findings:**

In this cohort study of 11 004 SNFs, urban, for-profit, and chain SNFs with lower staffing ratings and more Medicaid patients were more likely to change ownership. Ownership change was associated with short-term increases in emergency department visits but was not associated with hospital readmissions or community discharge rates.

**Meaning:**

These findings suggest that SNF ownership changes may be a symptom, rather than a cause, of lower quality care for short-stay patients.

## Teaser

This cohort study examines which skilled nursing facility characteristics are associated with changes in ownership and whether changes in skilled nursing facility ownership were associated with differences in short-stay patient outcomes.

## Introduction

Skilled nursing facilities (SNFs) were among the hardest hit places during the COVID-19 pandemic in the US, and as a result, the industry has come under significant public scrutiny.^1^ Understanding variations in patient safety and outcomes and incentivizing quality improvements in SNFs are increasingly urgent national priorities.^2^ The Biden Administration, the Centers for Medicare & Medicaid Services (CMS), and the National Committee on the Quality of Care in Nursing Homes have all recently recommended improving oversight of the SNF industry through a better understanding of SNF ownership structures.^2,3,4,5^ Private equity ownership of SNFs has also come under scrutiny; however, only approximately 11% of SNFs are owned by private equity firms.^6,7^

Changes in ownership for SNFs are frequent, with 3254 SNFs sold in the US between 2016 and 2021.^8,9^ In addition to potentially shifting funds away from patient care, SNF sales may further disrupt administrative and clinical processes, including staffing, admission practices, or discharge coordination. Two categories of patients in SNFs that may be impacted by changes in ownership are long-stay residents and short-stay patients admitted for rehabilitation after a hospital stay.^10^ Medicare covered postacute short stays for more than 1.2 million beneficiaries in 2020.^10^ While short-stay Medicare patients make up approximately 10% of SNF patients, Medicare provides a disproportionately large share of SNF revenue (mean, 17%), and ownership changes may shift a facility’s interest in admitting more profitable Medicare patients.^10,11^ Additionally, SNFs are subject to financial penalties or bonuses based on outcomes for short-stay patients through the SNF Value-Based Purchasing (VBP) program.^12^ Short-stay patient admissions and outcomes thus factor heavily into the financial picture of individual SNFs.

The impact of SNF changes in ownership on short-stay patient outcomes is unclear. Previous work has either focused on long-stay populations, included limited types of ownership changes, or lacked important outcomes. A 2013 study^13^ examined corporate structure and ownership of Texas facilities, finding few associations between ownership and assessed variables, such as nurse staffing and deficiencies on regulatory surveys, but did not examine patient-level outcomes. Other studies found mixed associations between changes in ownership and health outcomes for long-stay residents but only examined specific conversions from nonprofit to for-profit status or private equity acquisition, excluding short-stay patients and other sales that may increase the complexity of SNF ownership structures.^7,11,14^ Acquisition of SNFs by private equity firms between 2000 and 2017 was associated with increased costs, increased short-term mortality, and declines in mobility for short-stay Medicare patients.^15^ Outside of private equity acquisition, the impacts of SNF sales on short-stay patient outcomes have not been studied. Additionally, it is important to include short-stay outcomes that reflect patient experiences and aspects of care quality, such as discharge to the community, hospital readmissions, and emergency department (ED) visits after SNF discharge.^16^ Hospital readmissions are included in the SNF VPB program, and community discharge is an important metric of the quality-of-care coordination and rehabilitation and will be included in the SNF VBP program starting in October 2026.^12^

To improve transparency and facilitate research on associations between SNF ownership and quality, CMS released new data sets on SNF changes in ownership.^3^ This study leverages these data to examine associations between facility characteristics and SNF changes in ownership between 2016 and 2019 as well as associations between SNF changes in ownership and short-stay patient outcomes.

## Methods

This cohort study was deemed exempt from review and informed consent per the institutional review board of the University of Washington because all data are public and deidentified. We followed the Strengthening the Reporting of Observational Studies in Epidemiology (STROBE) reporting guideline. Our sample included all Medicare-certified and dually Medicare-Medicaid–certified SNFs operating in the US between 2016 and 2019 that had short-stay quality measure scores published in CMS Nursing Home Compare (NHC) claims-based quality measure public data files.

### Outcome Measures

Outcomes were NHC short-stay quality measures, which are risk-adjusted at the facility level. CMS reports 3 claims-based short-stay quality measures for SNFs: percentage of short-stay residents who were rehospitalized within 30 days of a nursing home admission (percentage readmitted),^16^ percentage of short-stay residents who have an outpatient ED visit (percentage ED visits), and percentage of short-stay residents who were successfully discharged to a community setting without readmission to a hospital or nursing home within 30 days (percentage community discharges).^16^ CMS defines community settings as private homes, assisted living facilities, or group homes, in contrast with inpatient hospitals, nursing homes, or other institutional settings.^16,17^ SNFs with higher quality have lower percentage readmitted and percentage ED visits and higher percentage community discharges outcomes.

Risk-adjusted short-stay quality scores are released quarterly but incorporate 4 quarters (Qs) of data into a 12-month mean score; therefore, quality scores reported from consecutive Qs will have 3 Qs of overlapping data. Because of this, differences between quality measures between 2 consecutive Qs actually represent the year-over-year difference in quality scores for a particular Q. For example, a Q4 2019 quality measure is the mean of quality scores from Q1 through Q4 in 2019, while a Q1 2020 quality measure is the mean of quality scores from Q2 to Q4 in 2019 and Q1 in 2020, meaning any difference in the quality measures for these 2 Qs is explained by the difference in quality scores between the specific quarters Q1 2019 and Q1 2020. Only the percentage readmitted and percentage ED visits measures were included in CMS files after Q1 2018 because these measures were not collected for inclusion in NHC 5-star ratings starting in 2018.^16^

### Change in Ownership

To identify SNFs that changed ownership, we used CMS SNF changes in ownership files,^3,18^ which include effective dates of all SNF sales, mergers, and consolidations starting in 2016. In changes in ownership files, ownership change reflects the transfer of the previous owner’s Medicare identification number and any outstanding Medicare debt to a new owner.^18^ We created indicators for the Q and year of ownership change between Q1 2016 and Q4 2019 and merged changes in ownership data with the NHC short-stay outcomes data.

### Facility Covariates

We included multiple facility-level characteristics from different public data sources. NHC files provided annual 5-star staffing ratings,^13^ SNF ownership (for-profit, nonprofit, or government), and Medicare-certified bed count to reflect facility size.^13^ We used Provider of Services files for urban vs rural location and state. We used LTCFocus^19^ files for annual status for the following variables: occupancy rate, chain affiliation, hospital-based vs freestanding facility, and percentage Medicaid patients.

### Statistical Analysis

First, we conducted a facility-level analysis using multivariable logistic regression to examine facility characteristics associated with changes in ownership. For the facilities that underwent changes in ownership, we used annual covariates from the year prior to the change in ownership as baseline characteristics for independent variables. For the facilities that did not undergo a change in ownership, we used data from the first year the SNFs appeared in the NHC files for baseline characteristics. Independent variables included facility covariates and state fixed effects to control for state-level market and regulatory factors.

Next, we used a controlled interrupted time series analysis to examine the effects of changes in ownership on the 3 facility-level short-stay patient outcomes: percentage readmitted, percentage ED visits, and percentage community discharges.^20^ Our treatment group was SNFs that underwent changes in ownership at any point between Q1 2016 and Q4 2019. The control group included SNFs operating in the US without an ownership change during the same time period. Similar to a standard difference-in-differences analysis, this approach allows us to estimate the differences in outcomes attributable to an ownership change, but also has the benefit of allowing for changes in ownership to occur at different time points throughout the study while adjusting for potential differences in trends from before ownership changes in quality between groups, thus not requiring the parallel trends assumption to be met.^21^ We did not assume parallel trends in quality a priori because previous reports found that SNFs with lower 5-star overall ratings and more deficiencies are more likely to undergo changes in ownership.^9,22^

For each outcome, we used a separate linear mixed effects regression model with a random intercept for facility to account for repeated measures for each SNF. We included the 5 fixed effects in our model. The first fixed effect analyzed was time, represented by Q for which the quality measures was reported (which included data from that Q as well as the 3 preceding it). This term estimates the longitudinal trend in the control group. The second fixed effect was a pre–ownership change indicator identifying SNFs that have not undergone change in ownership yet but will in a future Q. This term interacted with time estimates the difference in longitudinal trends between controls and the treatment group before the changes in ownership took place. The third fixed effect was an indicator identifying whether change in ownership occurred in the current or a prior Q. This indicator estimates the short-term outcome associated with ownership change, comparing the mean difference between quality scores in the Q the ownership change took place vs controls. The fourth fixed effect was a variable measuring how long since the change in ownership took place (only applicable after change in ownership). This variable, when interacted with the changes in ownership indicator, estimates the difference in long-term longitudinal trends after ownership change compared with the controls. The fifth fixed effect was facility characteristics that were used as adjustment variables in the model.

Finally, we conducted a sensitivity analysis to examine the additional associations with outcomes for SNFs specifically converting from nonprofit to for-profit status using the same analytical approach, with the treatment group defined as SNFs that converted profit status and the control group was SNFs that changed ownership but did not change profit status.

Analyses were completed with RStudio version 2022.07.02 (R Project for Statistical Computing). Results were considered statistically significant results at 2-sided α < .05. Data were analyzed from January 2016 through December 2019.

## Results

**Figure 1.**
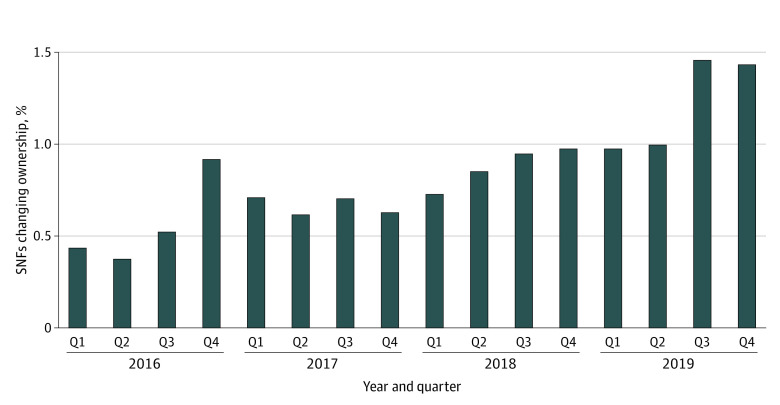
Skilled Nursing Facilities (SNFs) Changing Ownership Per Quarter (Q)

Of the 12 685 SNFs included in the NHC outcomes files, our final sample included 11 004 SNFs that were in operation throughout the study period and had complete facility characteristic data from all other sources. Table 1 includes descriptive statistics for baseline characteristics for SNFs in the final sample based on whether they did or did not change ownership. Of 11 004 SNFs, 1459 (13.26%) changed ownership in the 4 years of the study, with 534 (36.60%) of changes occurring in 2019 (Figure 1). Of 1459 SNFs changing ownership, 93 (6.37%) changed from nonprofit to for-profit ownership. Table 2 includes results from the multivariable logistic regression analysis for facility characteristics associated with changes in ownership.

**Table 1.** Descriptive Statistics for Skilled Nursing Facilities Operating Between 2016 and 2019 With Data on Short-Stay Patient Outcomes and Complete Data on Facility Characteristics

Characteristic	Facilities, No. (%) (N = 11 004)	
	Changed ownership (n = 1459)	Did not change ownership (n = 9513)
Ownership		
For-profit	1293 (88.62)	6692 (70.35)
Nonprofit	143 (9.80)	2270 (23.86)
Government	23 (1.58)	551 (5.79)
Rural	419 (28.72)	2751 (28.92)
Certified beds, mean (SD), No.	108.94 (48.01)	113.07 (60.72)
Occupancy rate, mean (SD), %	79.96 (13.82)	82.82 (13.35)
Hospital based	24 (1.64)	275 (2.89)
Chain	1016 (69.64)	5482 (57.63)
5-Star staffing rating, mean (SD)	2.91 (0.94)	3.20 (1.03)
Medicaid patients, mean (SD), %	66.92 (16.80)	61.32 (19.31)
CMS region		
Region 1 (Boston, Massachusetts)	63 (4.32)	723 (7.60)
Region 2 (New York, New York)	84 (5.76)	711 (7.47)
Region 3 (Philadelphia, Pennsylvania)	139 (9.53)	982 (10.32)
Region 4 (Atlanta, Georgia)	315 (21.59)	1678 (17.64)
Region 5 (Chicago, Illinois)	343 (23.51)	2032 (21.36)
Region 6 (Dallas, Texas)	147 (10.08)	800 (8.41)
Region 7 (Kansas City, Missouri)	98 (6.72)	1015 (10.67)
Region 8 (Denver, Colorado)	78 (5.35)	310 (3.26)
Region 9 (San Francisco, California)	159 (10.90)	971 (10.21)
Region 10 (Seattle, Washington)	33 (2.26)	291 (3.06)

**Table 2.** Logistic Regression Model of Odds of Skilled Nursing Facility Change in Ownership Based on Facility Characteristics

Characteristic	Odds ratio (95% CI)^a^	*P* value
Ownership		
For-profit	1 [Reference]	NA
Nonprofit	0.40 (0.32-0.49)	<.001
Government	0.26 (0.17-0.41)	<.001
Rural	0.86 (0.75-0.99)	.049
No. of certified beds, per 10-bed increase	0.97 (0.96-0.99)	<.001
Occupancy rate, per 10–percentage point increase	0.84 (0.80-0.88)	<.001
Hospital based	1.32 (0.83-2.08)	.24
Chain	1.38 (1.21-1.59)	<.001
5-Star staffing rating	0.85 (0.80-0.88)	<.001
Percentage Medicaid patients, per 10–percentage point increase	1.17 (1.13-1.22)	<.001

Abbreviation: NA, not applicable.

^a^ Models also adjusted for state fixed effects.

Larger SNFs with higher occupancy rates had lower odds of changes in ownership. Nonprofit and government SNFs had lower odds of undergoing changes in ownership compared with for-profit SNFs, we observed lower odds of undergoing changes in ownership in nonprofit SNFs (odds ratio [OR], 0.40; 95% CI, 0.32-0.49) and government SNFs (OR, 0.26; 95% CI, 0.17-0.41). SNFs that changed ownership had higher odds of being part of a chain compared with independent SNFs (OR, 1.38; 95% CI, 1.21-1.59). Chain SNFs had higher odds of changing ownership than nonchain SNFs (OR, 1.38; 95% CI, 1.21-1.59). Urban SNFs with lower occupancy rates (OR per 10–percentage-point decrease, 1.19; 95% CI, 1.14-1.25), larger Medicaid populations (OR per 10–percentage-point increase, 1.17; 95% CI, 1.13-1.22), and lower staffing ratings (OR per 1-star increase on staffing rating, 1.18; 95% CI, 1.14-1.25) had higher odds of changing ownership (Table 2).

**Figure 2.**
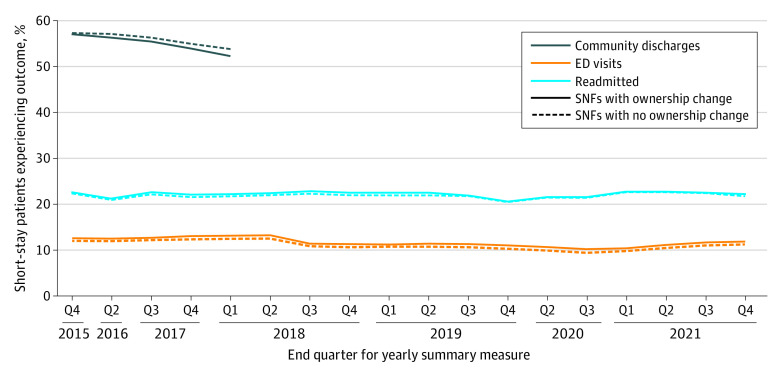
Short-Stay Outcomes Over Time in 11 004 Skilled Nursing Facilities (SNFs) in the United States ED indicates emergency department.

Figure 2 includes short-stay quality outcomes over time for SNFs that did and did not have changes in ownership. SNFs that had a change in ownership between January 2016 and December 2019 had lower quality scores throughout the study period compared with SNFs that never had a change in ownership. However, controlled interrupted time series results found that the only significant short-term outcome associated with change in ownership was a 0.32 (95% CI, 0.03-0.62) increase in the percentage ED visits outcome (Table 3). There were also no significant differences in longitudinal trends across the outcomes. Sensitivity analysis results demonstrated no significant short- or long-term differences in any of the quality outcomes for the facilities that underwent specific nonprofit to for-profit conversions (Table 3).

**Table 3.** Controlled Interrupted Time-Series Analysis Estimating Differences in Short-Stay Quality Outcomes for SNFs Undergoing a Change in Ownership vs Control SNFs That Did Not Undergo Ownership Changes

Quality outcome	Coefficient (95% CI)^a^	*P* value
**Percentage community discharge**		
Control group longitudinal trend	−1.61 (−1.75 to −1.47)	<.001
Pre–change in ownership difference in longitudinal trend between treatment and control SNFs	0.17 (−0.28 to 0.63)	.46
Post–change in ownership difference in outcome between treatment and control SNFs		
Short-term	−0.10 (−1.36 to 1.15)	.87
Long-term difference in longitudinal trends	−0.28 (−2.86 to 2.30)	.83
Nonprofit to for-profit conversion only		
Short-term	−0.07 (−5.19 to 5.06)	.98
Long-term	−3.85 (−14.33 to 6.65)	.47
**Percentage emergency department visits**		
Control group longitudinal trend	−0.61 (−0.64 to −0.58)	<.001
Pre–change in ownership difference in longitudinal trend between treatment and control SNFs	0.23 (0.12 to 0.34)	<.001
Post–change in ownership difference in outcome between treatment and control SNFs		
Short-term	0.32 (0.03 to 0.62)	.03
Long-term difference in longitudinal trends	−0.05 (−0.13 to 0.43)	.83
Nonprofit to for-profit conversion only		
Short-term	0.26 (−0.81 to 1.34)	.63
Long-term	−0.04 (−1.88 to 1.80)	.96
**Percentage hospital readmissions**		
Control group longitudinal trend	0.03 (0.003 to 0.07)	.04
Pre–change in ownership difference in longitudinal trend between treatment and control SNFs	0.51 (0.37 to 0.64)	<.001
Post–change in ownership difference in outcome between treatment and control SNFs		
Short-term	0.27 (−0.08 to 0.61)	.09
Long-term difference in longitudinal trends	0.13 (−0.46 to 0.71)	.67
Nonprofit to for-profit conversion only		
Short-term	−0.01 (−1.25 to 1.23)	.99
Long-term	−0.19 (−2.40 to 2.02)	.87

Abbreviation: SNF, skilled nursing facility.

^a^ Models also adjusted for state fixed effects, ownership, staffing ratings, percentage Medicaid patients, urban vs rural county, chain-affiliated vs independent status, hospital-based vs freestanding location, and occupancy rate.

## Discussion

In this cohort study, ownership changes were more common in urban, for-profit, and chain SNFs with lower staffing ratings, lower occupancy rates, and larger Medicaid populations. These results were consistent with previous research on SNF ownership changes and profit conversions in the 1990s and early 2000s,^9,11,22,23,24^ suggesting stable trends in factors associated with SNF sales prior to a new Medicare reimbursement system for SNFs that was implemented in 2019.^9,25^ Ownership changes may be motivated by a variety of factors, ranging from poor quality or financial instability to goals of improved efficiency and management.^11,22,26^ While SNFs with lower profit margins have also been shown to be more likely to undergo changes in ownership,^15,26,27^ cost report data were not included in this study. We included occupancy rates and Medicaid percentages for facility characteristics that are associated with lower margins and staffing variables to capture differences in care that may result from lower margins; however, direct associations among financial health of a SNF, changes in ownership, and patient outcomes warrant future research.

While quality ratings were lower throughout the study in the SNFs with changes in ownership and many of the facility characteristics that were associated with changes in ownership have been previously associated with worse outcomes for short- and long-stay patients,^28,29,30,31,32,33,34^ our interrupted time series analysis found minimal changes in short-stay outcomes that could be directly attributed to changes in ownership in general or to nonprofit to for-profit conversions. While SNFs with changes in ownership had worse outcomes compared with controls, these differences occurred throughout the study period (ie, both before and after a change in ownership occurred), and were thus not attributable to the change in ownership itself. These findings are consistent with a 2016 study^22^ that found no difference in nursing home quality as measured by deficiency rates after chain acquisition, which suggests that chains targeted SNFs with quality problems and those problems continued after acquisition. The finding that worse short-stay patient outcomes occurred in SNFs with changes in ownership group throughout the study period, combined with the finding that changes in ownership were more common in SNFs with characteristics that have previously been associated with lower quality, suggests that changes in ownership may be a symptom, rather than a cause, of lower quality.

We did find a statistically significant short-term increase of 0.32 percentage points in the percentage ED visits outcome for SNFs that underwent changes in ownership compared with controls, which is consistent with work by Braun et al^14^ that found 11.1% higher rates of ED visits for long-stay residents after private equity acquisition. However, the small magnitude of the difference in ED visits attributable to changes in ownership and the lack of difference for community discharge and readmissions are inconsistent with the study by Braun et al,^14^ which found 8.7% higher risk of hospitalizations after private equity acquisition. Smaller and fewer significant differences in our study may be due to including all forms of changes in ownership rather than just private equity acquisition, which carries significant financial pressures for SNFs and thus may have larger impacts on clinical and administrative processes that would impact patient outcomes compared with changes in ownership in general.^35^ Additionally, the risk-adjusted short-stay outcomes used in this study account for a larger variety of patient characteristics than the adjustment methods used by Braun et al.^14^ Robust risk adjustment would serve to bias differences between treatment and control SNFs toward the null. Finally, we adjusted for clinical and administrative variables, such as staffing, which may have larger impacts on care delivery, and therefore short-stay patient outcomes, than change in ownership on its own.^36,37^

Beyond adjusting for clinical and administrative variables that could impact care in our models, the nature of the ownership changes included in the changes in ownership files may have impacted the lack of association between ownership changes and patient outcomes in this study. Specifically, CMS requires SNFs to self-report transfers of greater than 5% ownership interest, which could include transactions between subsidiaries that already owned portions of the SNF; therefore, these transactions may not disrupt operations.^18^ It is also notable that many ownership changes in the changes in ownership data are between entities with very similar names, which could indicate restructuring within current subsidiaries rather than transfers of ownership to entirely different entities that could usher in more significant changes in administrative or clinical processes. With the current changes in ownership data, it is impossible to differentiate between SNF sales that may or may not disrupt patient care. Future work that examines administrator or staff turnover would be valuable in better identifying operational disruptions associated with SNF ownership changes.

### Limitations

This study has some limitations. Our study uses 2016 to 2019 data to establish associations of ownership change with short-stay outcomes prior to 2019 Medicare payment reform impacting SNFs and prior to the COVID-19 pandemic.^25^ SNF ownership changes continued into the pandemic,^8,9^ and research with more recent data is warranted to explore whether SNF sales may have been associated with patient outcomes during the public health emergency.^1^ Per the changes in ownership data, only ownership changes occurred in this time period, and no SNFs serving patients in the changes in ownership data set underwent mergers or consolidations as defined by CMS. However, as mentioned previously, changes in ownership data may not accurately reflect similar entities or adequately reflect private equity acquisition, which may have biased our results toward the null by undercounting ownership changes or profit conversions.^14,38^ CMS recently released plans to require and publish more accurate reporting of SNF ownership types,^4^ which will be important targets for future work. Additionally, the inability to separate unique quarters of data made timely impacts of changes in ownership difficult to isolate, as differences in quality measures from subsequent quarters represented a year-over-year change in quality scores for the latter Q, rather than a difference in the quality scores for the subsequent Qs. Furthermore, we did not have data about when a changes in ownership took place within a given Q, which could further impact the quality scores, particularly for the Q in which changes in ownership took place.

## Conclusions

In this longitudinal cohort study of 11 004 SNFs operating between 2016 and 2019, we found higher rates of ownership changes in SNFs with characteristics that have been historically associated with worse patient outcomes and worse short-stay patient outcomes in SNFs that underwent changes in ownership. However, SNF ownership changes were not independently associated with significant differences in most risk-adjusted short-stay patient outcomes when accounting for other facility characteristics. Thus, regulatory policies aimed only at limiting SNF sales may not have impacts on quality outcomes for short-stay patients. Efforts targeted toward improving clinical care and administrative processes, such as nurse and therapy staffing, may be more directly impactful for improving quality outcomes for patients in SNFs. Ultimately, SNFs that underwent changes in ownership in our sample had worse short-stay patient outcomes throughout the study period compared with SNFs that maintained the same ownership, suggesting that changes in ownership may be a symptom, not a cause, of lower quality for short-stay patients in SNFs.

## Notes

Supplement.
Data Sharing Statement

## References

[1] McGarry BE, Grabowski DC. Nursing homes and COVID-19: a crisis on top of a crisis. Ann Am Acad Pol Soc Sci. 2021;698(1):137-162. doi:10.1177/00027162211061509

[2] National Academies of Sciences Engineering and Medicine. The National Imperative to Improve Nursing Home Quality: Honoring Our Commitment to Residents, Families, and Staff. The National Academies of Sciences Engineering Medicine; 2022. doi:10.17226/26526 36198022

[3] Centers for Medicare & Medicaid Services. Skilled nursing facility change of ownership. Accessed June 24, 2022. https://data.cms.gov/provider-characteristics/hospitals-and-other-facilities/skilled-nursing-facility-change-of-ownership

[4] Department of Health and Human Services. Medicare and Medicaid programs; disclosures of ownership and additional disclosable parties information for skilled nursing facilities and nursing facilities: proposed rule. Accessed February 14, 2023. https://public-inspection.federalregister.gov/2023-02993.pdf

[5] The White House Briefing Room. Fact sheet: protecting seniors by improving safety and quality of care in the nation’s nursing homes. Accessed March 13, 2022. https://www.whitehouse.gov/briefing-room/statements-releases/2022/02/28/fact-sheet-protecting-seniors-and-people-with-disabilities-by-improving-safety-and-quality-of-care-in-the-nations-nursing-homes/

[6] Harrington C, Montgomery A, King T, Grabowski DC, Wasserman M. These administrative actions would improve nursing home ownership and financial transparency in the post COVID-19 period. *Health Affairs Forefront*. February 11, 2021. Accessed June 22, 2022. https://altarum.org/news/these-administrative-actions-would-improve-nursing-home-ownership-and-financial-transparency

[7] Huang SS, Bowblis JR. Private equity ownership and nursing home quality: an instrumental variables approach. Int J Health Econ Manag. 2019;19(3-4):273-299. doi:10.1007/s10754-018-9254-z 30357589

[8] Welch WP, Ruhter J, Bosworth A, Lew ND, Sommers BD. Changes of ownership of hospital and skilled nursing facilities: an analysis of newly-released CMS data. Accessed August 18, 2023. https://aspe.hhs.gov/reports/changes-ownership-hospital-skilled-nursing-facilities

[9] Assistant Secretary for Planning and Evaluation. Changes in ownership of skilled nursing facilities from 2016 to 2021: variations by geographic location and quality. Accessed November 13, 2022. https://aspe.hhs.gov/sites/default/files/documents/78aae3d6d528e77a729288746ccc2e84/changes-ownership-snf.pdf

[10] Medicare Payment Advisory Commission (MedPAC). Report to the Congress: Medicare payment policy. Accessed June 24, 2022. https://www.medpac.gov/document/march-2022-report-to-the-congress-medicare-payment-policy/

[11] Grabowski DC, Stevenson DG. Ownership conversions and nursing home performance. Health Serv Res. 2008;43(4):1184-1203. doi:10.1111/j.1475-6773.2008.00841.x 18355255 PMC2517264

[12] Centers for Medicare & Medicaid Services. Medicare program; prospective payment system and consolidated billing for skilled nursing facilities; updates to the Quality Reporting Program and Value-Based Purchasing Program for federal fiscal year 2023: requirements for long-term care facilities. *Federal Register*. August 3, 2022. Accessed August 18, 2023. https://www.federalregister.gov/documents/2022/08/03/2022-16457/medicare-program-prospective-payment-system-and-consolidated-billing-for-skilled-nursing-facilities

[13] Stevenson DG, Bramson JS, Grabowski DC. Nursing home ownership trends and their impacts on quality of care: a study using detailed ownership data from Texas. J Aging Soc Policy. 2013;25(1):30-47. doi:10.1080/08959420.2012.705702 23256557 PMC4825679

[14] Braun RT, Jung HY, Casalino LP, Myslinski Z, Unruh MA. Association of private equity investment in US nursing homes with the quality and cost of care for long-stay residents. JAMA Health Forum. 2021;2(11):e213817. doi:10.1001/jamahealthforum.2021.3817 35977267 PMC8796926

[15] Gupta A, Howell ST, Yannelis C, Gupta A. Does private equity investment in healthcare benefit patients: evidence from nursing homes. SSRN Electron J. Preprint posted online February 22, 2021. doi:10.2139/ssrn.3790212

[16] Abt Associates. Nursing home compare claims-based quality measure technical specifications. Accessed August 18, 2023. https://www.cms.gov/Medicare/Provider-Enrollment-and-Certification/CertificationandComplianc/Downloads/Nursing-Home-Compare-Claims-based-Measures-Technical-Specifications-April-2019.pdf

[17] Centers for Medicare and Medicaid Services. Quality Measures. Accessed August 18, 2023. https://www.cms.gov/medicare/quality-initiatives-patient-assessment-instruments/nursinghomequalityinits/nhqiqualitymeasures

[18] Centers for Medicare & Medicaid Services (CMS). Medicare Fee-For-Service provider enrollmentm—skilled nursing facility change of ownership: data guidance. Accessed December 12, 2022. https://data.cms.gov/sites/default/files/2022-10/SNF_CHOW_Data_Guidance_2022.09.30.pdf

[19] Brown University School of Public Health. LTCFocus public use data. Accessed August 23, 2023. https://ltcfocus.org/

[20] Lopez Bernal J, Cummins S, Gasparrini A. The use of controls in interrupted time series studies of public health interventions. Int J Epidemiol. 2018;47(6):2082-2093. doi:10.1093/ije/dyy135 29982445

[21] Fry CE, Hatfield LA. Birds of a feather flock together: comparing controlled pre-post designs. Health Serv Res. 2021;56(5):942-952. doi:10.1111/1475-6773.13697 34212387 PMC8522572

[22] Grabowski DC, Hirth RA, Intrator O, . Low-quality nursing homes were more likely than other nursing homes to be bought or sold by chains in 1993-2010. Health Aff (Millwood). 2016;35(5):907-914. doi:10.1377/hlthaff.2015.1042 27140998

[23] Bowblis JR. Ownership conversion and closure in the nursing home industry. Health Econ. 2011;20(6):631-644. doi:10.1002/hec.1618 21456048

[24] Castle NG. Nursing home closures, changes in ownership, and competition. Inquiry. 2005;42(3):281-292. doi:10.5034/inquiryjrnl_42.3.281 16355492

[25] Prusynski R. Medicare payment policy in skilled nursing facilities: lessons from a history of mixed success. J Am Geriatr Soc. 2021;69(12):3358-3364. doi:10.1111/jgs.17490 34569623 PMC8649036

[26] Lu LX, Lu SF. Does nonprofit ownership matter for firm performance: financial distress and ownership conversion of nursing homes. Manage Sci. 2022;68(7):5127-5145. doi:10.1287/mnsc.2021.4159

[27] Holmes JS. The effects of ownership and ownership change on nursing home industry costs. Health Serv Res. 1996;31(3):327-346. 8698588 PMC1070122

[28] Prusynski RA, Frogner BK, Dahal AD, Skillman SM, Mroz TM. Skilled nursing facility characteristics associated with financially motivated therapy and relation to quality. J Am Med Dir Assoc. 2020;21(12):1944-1950.e3. doi:10.1016/j.jamda.2020.04.008 32513557

[29] Lepore M, Leland NE. Nursing homes that increased the proportion Of medicare days saw gains in quality outcomes for long-stay residents. Health Aff (Millwood). 2015;34(12):2121-2128. doi:10.1377/hlthaff.2015.0303 26643633 PMC5369416

[30] Zuckerman RB, Wu S, Chen LM, Joynt Maddox KE, Sheingold SH, Epstein AM. The five-star skilled nursing facility rating system and care of disadvantaged populations. J Am Geriatr Soc. 2019;67(1):108-114. doi:10.1111/jgs.15629 30339726

[31] Bos A, Boselie P, Trappenburg M. Financial performance, employee well-being, and client well-being in for-profit and not-for-profit nursing homes: a systematic review. Health Care Manage Rev. 2017;42(4):352-368. doi:10.1097/HMR.0000000000000121 28885990

[32] Comondore VR, Devereaux PJ, Zhou Q, . Quality of care in for-profit and not-for-profit nursing homes: systematic review and meta-analysis. BMJ. 2009;339(7717):b2732. doi:10.1136/bmj.b2732 19654184 PMC2721035

[33] O’Neill C, Harrington C, Kitchener M, Saliba D. Quality of care in nursing homes: an analysis of relationships among profit, quality, and ownership. Med Care. 2003;41(12):1318-1330. doi:10.1097/01.MLR.0000100586.33970.58 14668664

[34] Hillmer MP, Wodchis WP, Gill SS, Anderson GM, Rochon PA. Nursing home profit status and quality of care: is there any evidence of an association? Med Care Res Rev. 2005;62(2):139-166. doi:10.1177/1077558704273769 15750174

[35] Gandhi A, Anderson U, Song Y, Fowler S, Upadrashta P, Fuqua D. Private equity, consumers, and competition. SSRN. Preprint posted online May 16, 2023. doi:10.2139/ssrn.3626558

[36] Mukamel DB, Saliba D, Ladd H, Konetzka RT. Associations between daily nurse staffing levels and daily hospitalizations and ED visits in nursing homes. J Am Med Dir Assoc. 2022;23(11):1793-1799.e3. doi:10.1016/j.jamda.2022.06.030 35948066 PMC9955393

[37] Mukamel DB, Saliba D, Ladd H, Konetzka RT. Association of staffing instability with quality of nursing home care. JAMA Netw Open. 2023;6(1):e2250389. doi:10.1001/jamanetworkopen.2022.50389 36626170 PMC9856742

[38] Braun RT, Williams D, Stevenson DG, . The role of real estate investment trusts in staffing US nursing homes. Health Aff (Millwood). 2023;42(2):207-216. doi:10.1377/hlthaff.2022.00278 36696597

