# Error in Conflicts of Interest

**DOI:** 10.1001/jamanetworkopen.2023.41008

**Published:** 2023-10-18

In the Original Investigation titled “Skilled Nursing Facility Changes in Ownership and Short-Stay Medicare Patient Outcomes,”^1^ published September 19, 2023, the Conflict of Interests Disclosures incorrectly reported that Dr Humbert received grant funding from the National Institute on Aging, but this was the grant funding for the study, and all authors received this funding. This article has been corrected to include information on the grant that funded the study and the role of the funder in the Article Information.^1^
