# Supplementary material for: Skilled Nursing Facility Changes in Ownership and Short-Stay Medicare Patient Outcomes
Source: JAMA Netw Open. 2023 Sep 19;6(9):e2334551. doi: 10.1001/jamanetworkopen.2023.34551 (PMC10509722; doi:10.1001/jamanetworkopen.2023.34551)
Supplement: Supplement. — Data Sharing Statement [file jamanetwopen-e2334551-s001.pdf]

Data Sharing Statement

Prusynski. Skilled Nursing Facility Changes in Ownership and Short-Stay Medicare Patient Outcomes. *JAMA Netw Open*. Published September 19, 2023.
doi:10.1001/jamanetworkopen.2023.34551

Data
Data available: No

Additional Information
Explanation for why data not available: Data are provided by CMS through a data use agreement with the authors that does not allow sharing of research-identifiable files.
